# Assessment of Variability in the SOMAscan Assay

**DOI:** 10.1038/s41598-017-14755-5

**Published:** 2017-10-27

**Authors:** Julián Candia, Foo Cheung, Yuri Kotliarov, Giovanna Fantoni, Brian Sellers, Trevor Griesman, Jinghe Huang, Sarah Stuccio, Adriana Zingone, Bríd M. Ryan, John S. Tsang, Angélique Biancotto

**Affiliations:** 1 Trans-NIH Center for Human Immunology, Autoimmunity, and Inflammation, National Institutes of Health, Bethesda, MD 20892 USA; 2 Laboratory of Immunoregulation, National Institute of Allergy and Infectious Diseases, National Institutes of Health, Bethesda, MD 20892 USA; 3 Laboratory of Human Carcinogenesis, Center for Cancer Research, National Cancer Institute, National Institutes of Health, Bethesda, MD 20892 USA; 4 Systems Genomics and Bioinformatics Unit, Laboratory of Systems Biology, National Institute of Allergy and Infectious Diseases, National Institutes of Health, Bethesda, MD 20892 USA

## Abstract

SOMAscan is an aptamer-based proteomics assay capable of measuring 1,305 human protein analytes in serum, plasma, and other biological matrices with high sensitivity and specificity. In this work, we present a comprehensive meta-analysis of performance based on multiple serum and plasma runs using the current 1.3 k assay, as well as the previous 1.1 k version. We discuss normalization procedures and examine different strategies to minimize intra- and interplate nuisance effects. We implement a meta-analysis based on calibrator samples to characterize the coefficient of variation and signal-over-background intensity of each protein analyte. By incorporating coefficient of variation estimates into a theoretical model of statistical variability, we also provide a framework to enable rigorous statistical tests of significance in intervention studies and clinical trials, as well as quality control within and across laboratories. Furthermore, we investigate the stability of healthy subject baselines and determine the set of analytes that exhibit biologically stable baselines after technical variability is factored in. This work is accompanied by an interactive web-based tool, an initiative with the potential to become the cornerstone of a regularly updated, high quality repository with data sharing, reproducibility, and reusability as ultimate goals.

## Introduction

SOMAscan^1^ is a highly multiplexed, aptamer-based assay optimized for protein biomarker discovery, which is made possible by the simultaneous measurement of a broad range of protein targets. This assay, which in the current 1.3k version measures 1,305 human protein analytes, has proved successful in the identification of biomarker signatures in a variety of recent biomedical applications, ranging from non-small cell lung cancer^2–4^ and Alzheimer’s disease^5,6^ to cardiovascular disease^7–9^ and inflammatory bowel disease^10^, among others. Furthermore, the large number of simultaneous measurements has also enabled proteomics-based genetic association studies to systematically capture associations across multiple chromosome locations^11^, leading to a genome-proteome network that may provide a basis for novel approaches to pharmaceutical and diagnostic applications. Due to evolutionary conservation, SOMAscan has proven useful in some applications to non-human species as well, as shown e.g. in recent mouse studies^12,13^.

The basis of the SOMAscan Assay relies upon a new generation of protein-capture *Slow Offrate Modified Aptamer* (SOMAmer) reagents^14^. Using these reagents, SOMAscan is able to comparatively evaluate protein abundance in a volume of 50 *μ*l of serum, plasma, or other biological matrices. For a previous version of the SOMAscan Assay involving 813 SOMAmers, studies of reproducibility in serum and plasma dating back to 2010 reported an overall intra- and interplate median coefficient of variation (CV) of ~5%^1^. However, for all the interest that this promising technology has provoked, no efforts have been devoted so far to create an up-to-date, comprehensive, publicly available resource to characterize the CVs of individual SOMAmers. Even more crucially, the translation of these CV values into an operational assessment of the assay’s technical variability (e.g. by informing expected fold-change disparities of replicate measurements and, thus, by enabling the rigorous analysis of statistical significance of interventions and/or disease progression) is altogether missing. Moreover, also lacking is a comparison of different data processing and normalization procedures typically used on SOMAscan datasets, which can significantly affect the reported results.

In this context, the goal of our work is to present a comprehensive meta-analysis of performance across multiple plates in serum and plasma with the current 1.3 k Assay, as well as serum samples using the previous 1.1 k Assay. Firstly, we assess different normalization procedures and point out caveats in which a departure from standard strategies is needed to avoid increasing plate effects, while illustrating these observations with data from a multiplate longitudinal vaccination study. Secondly, we present a meta-analysis by using calibrator replicates as probes to assess the intraplate variability and signal-over-background intensity of each SOMAmer in the SOMAscan Assay. Thirdly, we incorporate the measured CVs into a theoretical model of statistical variability, which provides an interpretation of intra- and interplate variability of calibrator and quality control samples in terms of fold change probability distributions. By utilizing these distributions as reference null models, SOMAscan users will be able to perform rigorous statistical tests of significance in intervention studies and clinical trials, as well as quality control within and across laboratories. Finally, we explore the issue of assessing biological vs. technical variability of intra-subject baseline measurements in a cohort of healthy individuals and provide a list of stability measurements for all SOMAmers in the 1.1 k Assay, which represents a valuable reference for future studies.

Jointly with this paper, we are making available an interactive web-based tool permanently hosted by the National Institutes of Health^15^, which will enable SOMAscan researchers full access to the results of our meta-analysis. As the first effort to build a SOMAscan resource for the benefit of the user community at large, this initiative has the potential to grow organically through the contributions of other users of this technology and serve as the starting point towards regularly updated, high quality repositories with data sharing, reproducibility, and reusability as ultimate goals.

## Methods

### Experimental

Proteomic profiles were characterized using the SOMAscan Assay (SomaLogic, Inc.; Boulder, CO, USA) at the Trans-NIH Center for Human Immunology, Autoimmunity, and Inflammation (CHI), National Institutes of Health (Bethesda, MD, USA). The basis of SOMAscan is built on the use of a new generation of protein-capture *Slow Offrate Modified Aptamer* (SOMAmer) reagents^14^. Using these reagents, the SOMAscan Assay is able to comparatively evaluate protein abundance in 50 *μ*l of serum, plasma, or other biological matrices. Generated by a technique referred to as *Selected Evolution of Ligands by Exponential Enrichment* (SELEX), the current 1.3 k Assay consists of 1,305 SOMAmer reagents selected against a variety of human proteins (47 % secreted proteins, 28 % extracellular domains, 25 % intracellular proteins) that belong to broad biological subgroups including receptors, kinases, cytokines, proteases, growth factors, protease inhibitors, hormones, and structural proteins.

For serum and plasma samples, SOMAmer reagents are binned into three separate groups according to the expected endogenous abundance of each SOMAmer’s cognate protein in typical human samples. Each SOMAmer reagent exists in only one of the three groupings. Serum and plasma samples (including controls) are then diluted into three concentrations (0.005%, 1%, and 40%) in order to create separate groups for high, medium, and low abundance proteins, respectively. Through this separation, the SOMAscan assay is able to quantify proteins across a dynamic range spanning more than 8 orders of magnitude. The diluted samples are then incubated with the dilution-specific SOMAmers.

Runs in the current 1.3 k Assay were performed semi-automatically with a Tecan Freedom Evo 200 *High Throughput System* (HTS), which utilizes 96— well plates. In this work, we also present serum runs performed manually with the former 1.1 k Assay using 32— well plates. Supplementary Figure 1(a,b) shows Venn diagram comparisons between the two assays based on aptamer sequence (“SeqId”) and target analyte, respectively. For a total of 1,061 SOMAmers, the aptamer sequence remained unchanged. However, for 60 SOMAmer targets, the aptamer was replaced to improve binding affinity and specificity. The case study presented below in the Results Section presents 1.1 k Assay data from one serum run performed by SomaLogic with a Beckman BioMek Fx HTS. The total number of samples analyzed is 2,624.

The typical SOMAscan plate design includes buffer wells (no sample added), quality control (QC_SOMAscan) and calibrator samples provided by SomaLogic. Quality control and calibrators are pooled samples composed of the same matrix as the biological samples being measured in the plate. Usually, 2 sets of quality control samples are run in duplicate in each plate. Since the intended purpose of these samples is to assess the quality of measurements obtained from one single plate, quality control samples may vary from plate to plate. Also, 5 to 7 replicate calibrator samples are included in each plate with the purpose of normalization across plates. The calibrator consists of a common pooled sample used across a large number of runs; however, when that calibrator lot is depleted, SomaLogic must switch to a different calibrator lot. In addition to these, we have added bridge samples (QC_CHI) to our plate designs in serum and plasma, which we typically run in quadruplicate in each plate and keep consistent across all runs in the 1.3k Assay. Our serum QC_CHI consists of 17 (8 male, 9 female) pooled samples from healthy donors of median age 35 (Q1 = 28.5, Q3 = 54.5) years. For our plasma QC_CHI, we pooled 21 (10 male, 11 female) samples from healthy donors of median age 57 (Q1 = 37, Q3 = 61.5) years. Table 1 presents summary statistics of all serum and plasma runs performed at CHI between January 2015 and April 2017, all of which were analyzed in this paper. The table includes, in parentheses, the breakdown in terms of (*n*_*rep*_ × *n*_*pl*_), where *n*_*rep*_ is the number of replicates per plate and *n*_*pl*_ is the number of plates.

**Table 1:** Summary statistics of all runs analyzed in this paper.

	Serum 1.3k (HTS)	Plasma 1.3 k (HTS)	Serum 1.1 k (Manual)
Plates	15	8	11
Buffer	19 (1 × 13, 3 × 2)	10 (1 × 7, 3 × 1)	11 (1 × 11)
Calibrators	101 (7 × 13, 5 × 2)	54 (7 × 7, 5 × 1)	55 (5 × 11)
QC_SOMAscan	57 (2 × 27, 3 × 1)	31 (2 × 14, 3 × 1)	22 (2 × 11)
QC_CHI	59 (4 × 14, 3 × 1)	28 (4 × 7)	0

In accordance with SOMAscan’s change log from December 2016, we removed the following 5 SOMAmers throughout: Alkaline phosphatase, tissue-nonspecic isozyme (SeqId 2795-23, UniProt P05186), Complement C1s subcomponent (SeqId 3590-8, UniProt P09871), Desmoglein-2 (SeqId 5071-3, UniProt Q14126), Reticulon-4 (SeqId 5118-74, UniProt Q9NQC3), Tumor necrosis factor receptor super-family member 25 (SeqId 5073-30, UniProt Q93038).

### Data Normalization Procedures

Raw data, as obtained after slide feature aggregation from slide-based hybridization microarrays, exhibit intraplate nuisance variance due to differences in loading volume, leaks, washing conditions, etc, which are then compounded with batch effects across plates. SomaLogic’s guidelines for data processing encompass three sequential levels of normalization, namely *Hybridization Control Normalization* (Hyb) followed by *Median Signal Normalization* (Hyb.MedNorm) and *Interplate Calibration* (Hyb.MedNorm.Cal), as explained below.

Hybridization Control Normalization is designed to adjust for nuisance variance on the basis of individual wells. Based on *n*_*HCE*_ = 12 *Hybridization Control Elution* (*HCE*) spike-ins, the observed *Relative Fluorescence Intensities* (RFUs) are compared to reference values and the scale factor for the *i*th sample is determined as

1 $$S{F}_{i}={\rm{median}}{\{\frac{RF{U}_{\alpha }^{HCE,ref}}{RF{U}_{i\alpha }^{HCE,obs}}\}}_{\alpha =\mathrm{1,...,}{n}_{HCE}}.$$

Notice that this normalization step is performed independently for each sample; once the scale factor is determined, all SOMAmer RFUs for the sample are multiplied by that scale factor. Formerly, hybridization scale factors were pinned to an external reference, but under current recommendations from SomaLogic, reference values are determined by the median across the plate, i.e.

2 $$RF{U}_{\alpha }^{HCE,ref}\equiv \text{median}\{RF{U}_{i\alpha }^{HCE,obs}{\}}_{i=1,\mathrm{...,}{n}_{s}}.$$

Naturally, this procedure is more accurate in HTS runs, which use larger plates (*n*_*s*_ = 96) than manual runs (*n*_*s*_ = 32). In this paper, Hyb normalization is performed using the intraplate reference method throughout.

Median Signal Normalization is an intraplate normalization procedure performed within wells of the same sample class (i.e. separately for buffer, each QC type, calibrator, and biological samples) and within SOMAmers of the same dilution grouping (i.e. 0.005%, 1%, and 40%). It is intended to remove sample-to-sample differences in total RFU brightness that may be due to differences in overall protein concentration, pipetting variation, variation in reagent concentrations, assay timing, and other sources of variability within a group of otherwise comparable samples. Since RFU brightness differs significantly across SOMAmers, median signal normalization proceeds in two steps. First, the median RFU of each SOMAmer is determined (across all samples in the same sample class) and sample RFUs are divided by it. The ratio corresponding to the *i*th sample and *α*th SOMAmer is thus given by

3 $${r}_{i\alpha }^{gd}=RF{U}_{i\alpha }/\text{median}{\{RF{U}_{j\alpha }\}}_{j\mathrm{=1,...,}{n}_{s}^{g}},$$

where indices *g* and *d* denote sample and SOMAmer dilution groupings, respectively. Then, the scale factor associated with the *i*t*h* sample is determined as the inverse of the median ratio for that sample across all SOMAmers in the dilution group:

4 $$S{F}_{i}^{gd}=1/\text{median}\{{r}_{i\alpha }^{gd}{\}}_{\alpha \mathrm{=1,...,}{n}_{SOMAmer}^{d}}.$$

To median-normalize the *i*th sample, then, all its SOMAmer RFUs in the same dilution group are multiplied by this scale factor.

Calibration Normalization is an interplate procedure performed separately on each SOMAmer, which is applied to all the samples in a plate. No protein spikes are added to the calibrator; the procedure relies solely on the endogenous levels of each analyte within the set of calibrator replicates. For the *α*th SOMAmer,

5 $$S{F}_{\alpha }=RF{U}_{\alpha }^{Cal,ref}/\text{median}\{RF{U}_{i\alpha }^{Cal,obs}{\}}_{i\mathrm{=1,...,}{n}_{Cal}}.$$

Calibration scale factors may also be pinned to an external reference, but here we set the arbitrary reference value $RF{U}_{\alpha }^{Cal,ref}\equiv 1$ for all SOMAmers throughout.

Finally, due to issues of plate bias discussed in the Results Section below, in this work we introduce an alternative interplate normalization procedure (Hyb.Cal) in which the calibration normalization step is applied after performing median signal normalization only on the calibrators, i.e. by skipping the MedNorm step on samples other than calibrators.

### Computational

This paper is accompanied by a web-based tool^15^ written with the R package Shiny, which features an interactive interface to generate summary statistics, fold change probability distributions, and critical value distributions for the HTS 1.3 k Assay (serum and plasma) and manual 1.1 k Assay (serum). Moreover, an upload form is available for SOMAscan users to contribute their calibrator and quality control samples. New data contributions will be processed by a back-end pipeline to update the tool at regular intervals.

### Data Availability

The datasets generated during and/or analyzed during the current study are available in the Open Science Framework repository, osf.io/x3jsq

### Ethical approval and informed consent

All clinical protocols were conducted in accordance with Declaration of Helsinki principles and were approved by the competent institutional review boards, as specified below. Written informed consent was obtained from all subjects. For the adenovirus Type 4-Influenza H5 recombinant vaccine study, blood samples were collected after written informed consent under protocols approved by the Institutional Review Board of the National Institute of Allergy and Infectious Diseases at the National Institutes of Health. For the non-small-cell lung cancer study, blood samples were collected after written informed consent under protocols approved by the Institutional Review Board of the National Cancer Institute at the National Institutes of Health and the Institutional Review Board of the University of Maryland. For the H1N1 influenza vaccine study, blood samples were collected after written informed consent and under protocols approved by the Institutional Review Board of the National Heart, Lung, and Blood Institute at the National Institutes of Health.

## Results

### Assessment of Data Normalization Procedures

As explained above in the Methods Section, SomaLogic’s guidelines for data processing encompass three sequential levels of normalization, namely *Hybridization Control Normalization* (Hyb) followed by *Median Signal Normalization* (Hyb.MedNorm) and *Interplate Calibration* (Hyb.MedNorm.Cal). Additionally, in this work we also consider the Hyb.Cal method, which skips the intermediate MedNorm step for all sample types except calibrators. Our robot-assisted *High Throughput System* (HTS) setup includes our own serum and plasma bridge samples, QC_CHI, which were pooled from multiple healthy donors and are typically run in quadruplicate in each plate across all runs in the 1.3k Assay. Since these bridge samples are not used in the calibration process, they allow us to comparatively assess different normalization procedures.

**Figure 1:**
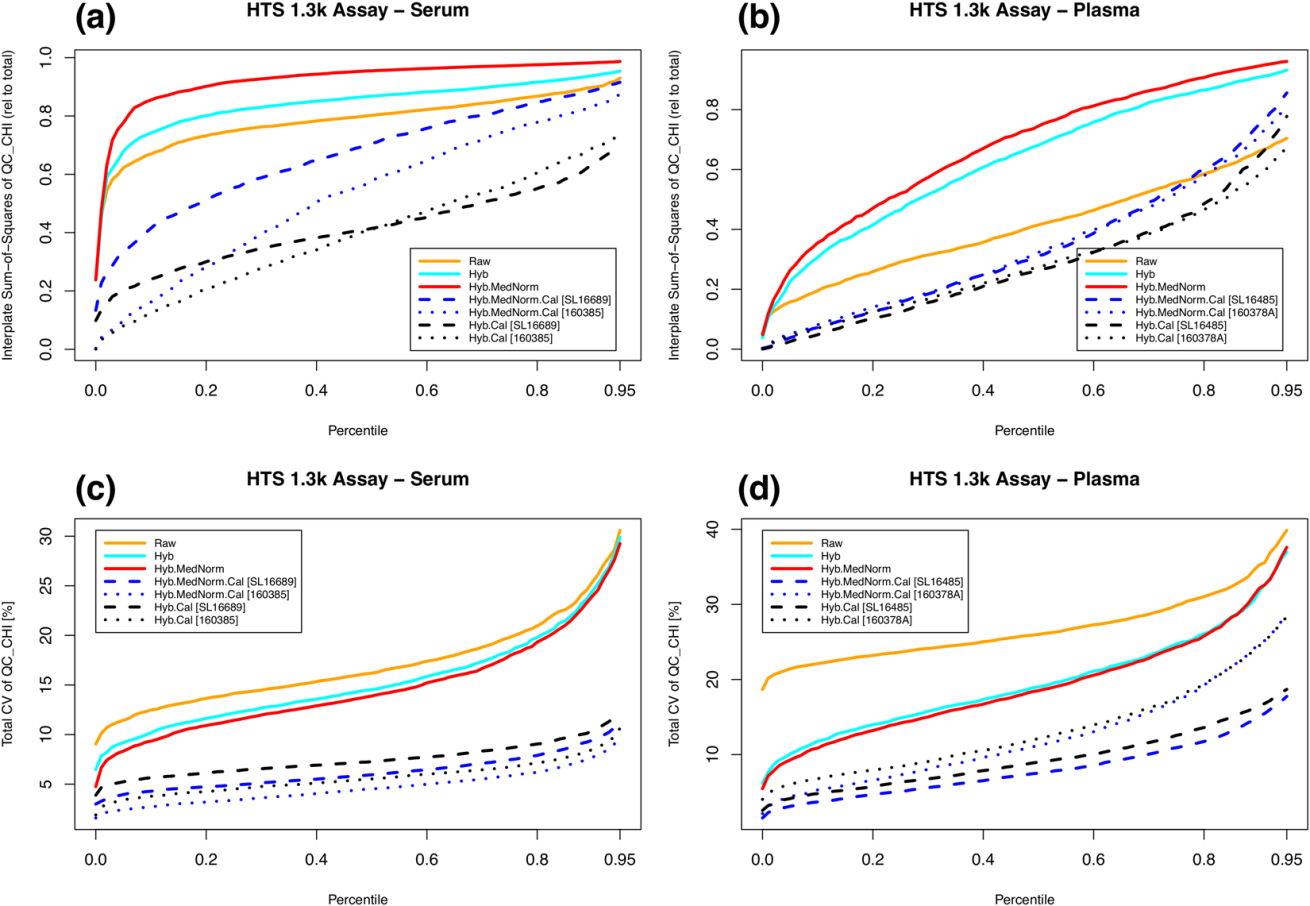
Assessment of data normalization for the HTS 1.3 k Assay. Top panels: Percentile distributions (across all SOMAmers) of the interplate Sum-of-Squares (relative to total SoS) for the bridge sample QC_CHI in (**a**) serum and (**b**) plasma. Bottom panels: Percentile distributions (across all SOMAmers) of the total CV for the bridge sample QC_CHI in (**c**) serum and (**d**) plasma.

In order to assess plate effects under different normalizations, we performed a one-way ANOVA to partition the total Sum-of-Squares (SoS) into interplate and residual components. The ratio of interplate SoS relative to the total SoS is a measure of inter- vs intra-plate variability. Figure 1(a,b) shows the percentile distribution (across all SOMAmers) of the interplate Sum-of-Squares (SoS) relative to the total SoS for the bridge sample QC_CHI in serum and plasma, respectively. In serum, we observe that interplate variability dominates the raw data (*SoS* > 0.5) for most SOMAmers. After Hyb and Hyb.MedNorm, the relative interplate SoS grows even larger, which is expected from the fact that these are intraplate normalization methods. Adding calibration, the relative interplate SoS is significantly lowered. Hyb.Cal normalization, which skips the MedNorm step, naturally leads to the lowest relative interplate variability. In plasma, the relative interplate SoS is comparatively lower, but the progression with normalization procedures is qualitatively the same as in serum. Figure 1(c,d) shows the percentile distribution (across all SOMAmers) of the total CV for the bridge sample QC_CHI in serum and plasma, respectively. The coefficient of variation (in percent units) is defined as *CV* ≡ 100 *σ*/*μ*, here and throughout. Although each step in the normalization sequence (from Raw to Hyb.MedNorm.Cal) is found to reduce overall variability, the CV distributions are most significantly lowered after the interplate calibration step. By skipping the Median Signal Normalization (MedNorm) step, CVs obtained from Hyb.Cal appear somewhat higher than those from Hyb.MedNorm.Cal. The MedNorm step is intended to remove sample-to-sample differences in total RFU brightness that may be due to nuisance factors, and works well in scenarios where samples are highly homogeneous within and across plates, as is indeed the case of bridge sample repeats. However, when used in the context of biological samples, which are inherently more heterogeneous, MedNorm may actually increase variability and plate bias. This issue, indeed, is observed in Supplementary Figure 2, where we analyzed the CV percentile distributions of 29 interplate duplicates of serum samples from early-stage non-small-cell lung cancer patients and heavy smoker donor controls run manually in the 1.1 k Assay. The lowest variability corresponds to Hyb.Cal.

**Figure 2:**
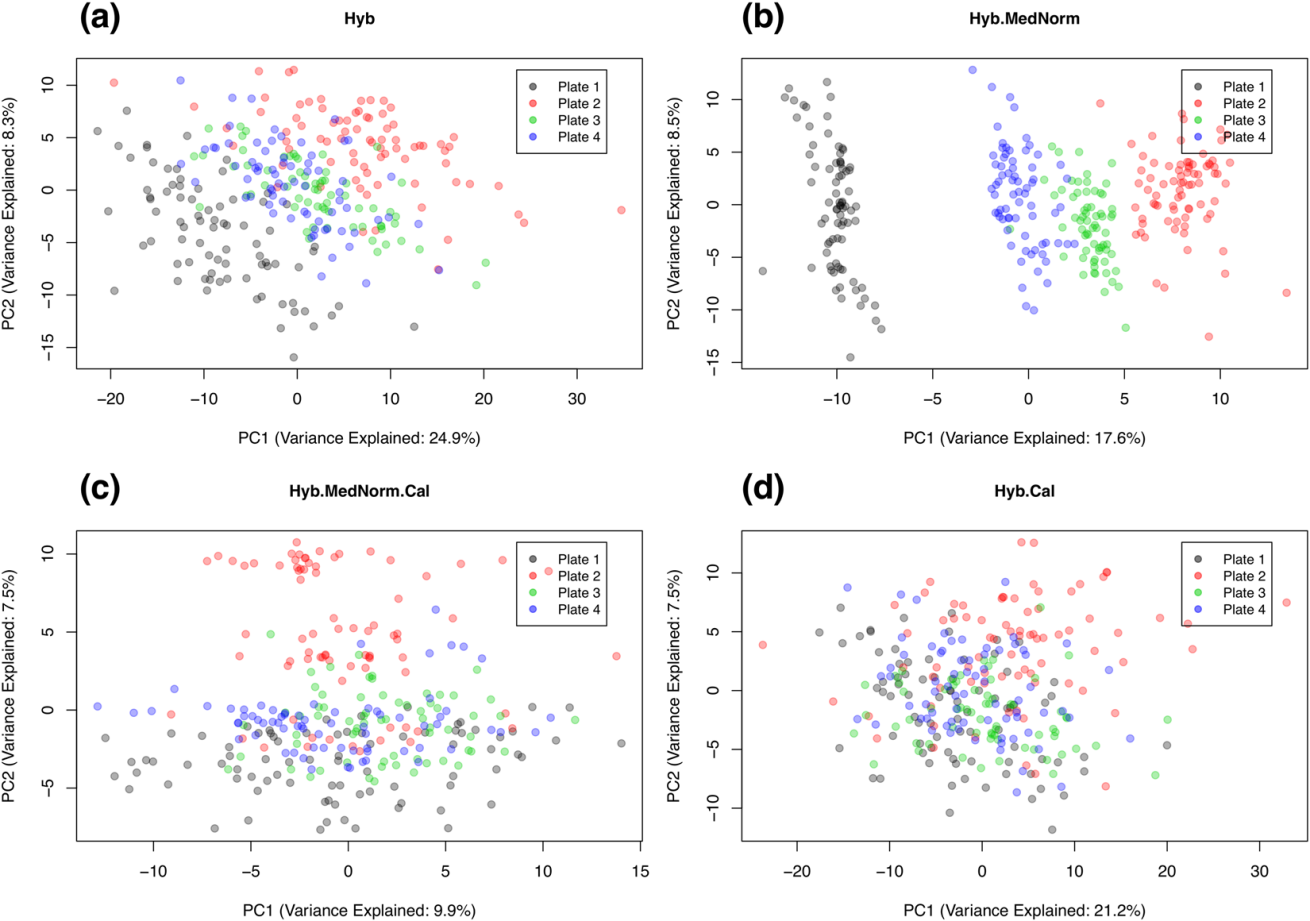
Principal component analysis of serum samples from a multiplate longitudinal study to explore response to an Adenovirus Type 4-Influenza H5 recombinant vaccine. Different normalizations are compared, as indicated. Data were generated with the HTS 1.3 k Assay.

In order to further investigate this issue, let us consider data from a multiplate longitudinal study designed to uncover correlates to the response to an Adenovirus Type 4-Influenza H5 recombinant vaccine. For the scope of this paper, data have been fully anonymized and used solely in the context of exploring technical variability across normalizations. Serum samples were obtained at multiple timepoints (ranging from 4 to 18 per subject, with a median of 9) over 33 healthy human subjects, which were randomized across plates. Since the characterization of the longitudinal progression of each individual is a primary endpoint of the study, all the timepoints from each subject were included in the same plate. Figure 2 shows the principal component analysis (PCA) of the data obtained using different normalizations. Each datapoint represents one sample, colored by plate. Since all 11 timepoints from one subject appeared as outliers in all PCA plots regardless of normalization, this outlier individual was removed from the analysis. Axes represent the top two principal components, in which the corresponding percent values of variance explained are indicated. From these results, it is evident that the MedNorm step, far from reducing plate effects, actually enhanced them. Even after performing interplate calibration, Hyb.MedNorm.Cal still shows a clearly visible plate-dependent bias. By skipping the MedNorm step, Hyb.Cal shows much less evidence of plate effects.

**Figure 3:**
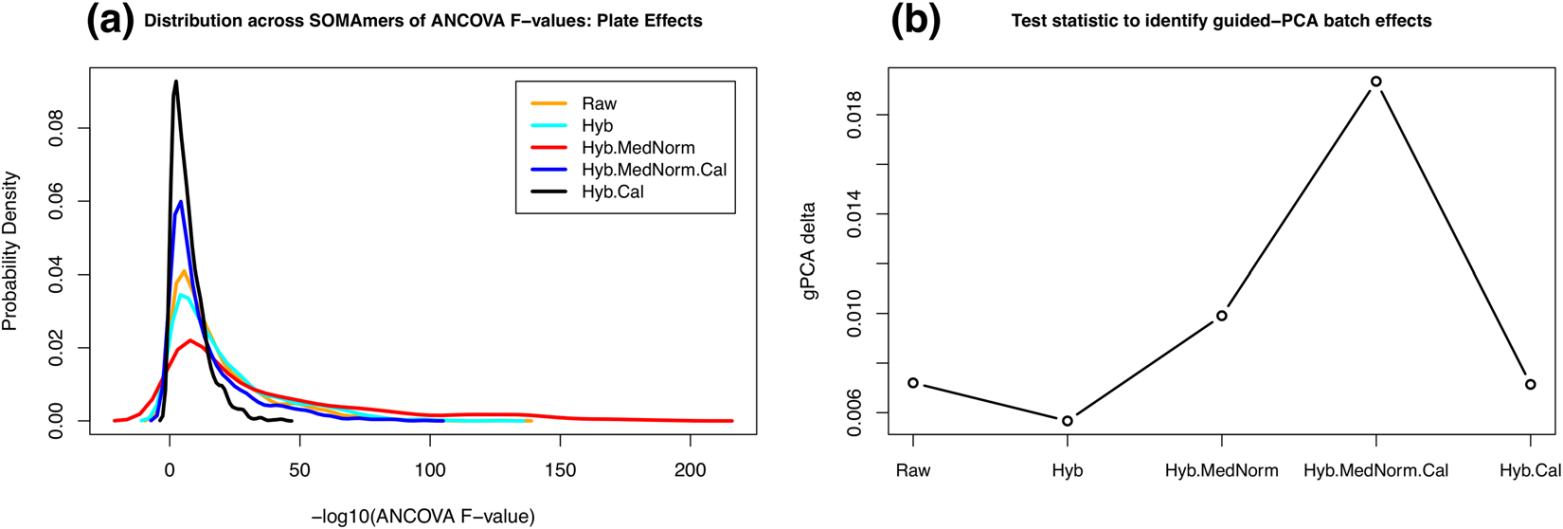
Quantitative assessments of plate effects: (**a**) Distribution of ANCOVA F-values across all SOMAmers; (**b**) guided-PCA delta statistic. Data were generated with the HTS 1.3 k Assay.

Following these qualitative observations, Fig. 3 offers results from two different quantitative assessments of plate effects. For each normalization and SOMAmer, we performed an ANCOVA test to model plate, sex, and age effects on the observed RFU. The F-value associated with each independent variable tests the null hypothesis (*H*_0_: no association between RFU and the independent variable). Figure 3(a) shows the distribution of F-values associated with plate effects across all SOMAmers for each normalization scheme, where we observe that Hyb.Cal yields the narrowest distribution, whereas Hyb.MedNorm the widest one. The top panels in Supplementary Figure 3 show ANCOVA F-value distributions for plate, sex and age, respectively. For sex and age, since interplate calibration is accounted for by the plate variable in the ANCOVA model, the Hyb and Hyb.Cal distributions are identical, as well as the Hyb.MedNorm and Hyb.MedNorm.Cal distributions. By comparing the range of F-values spanned, we observe that the primary factor of known variability is due to plate effects, while demographics play a lesser role. Further evidence is provided by the bottom panels of Supplementary Figure 3, which show the percentile sum-of-squares distribution across all SOMAmers of plate, sex, and age, respectively, relative to the total SoS. Additionally, Fig. 3(b) shows the so-called guided-PCA delta, proposed recently as a new statistic for identifying batch effects^16^. According to this statistic, plate effects increase significantly with median normalization and Hyb.Cal clearly outperforms Hyb.MedNorm.Cal among interplate normalization methods.

In summary, our meta-analysis strongly suggests that intraplate hybridization (Hyb) and interplate calibration (Cal) are two important normalization procedures to reduce nuisance variability. Intraplate median signal normalization (MedNorm) is a useful procedure to adjust for overall brightness differences across similar samples, such as calibrators, bridge and quality control intraplate repeats. However, intraplate median signal normalization might increase plate bias. This issue is particularly evident when applied to a heterogeneous set of samples, e.g. mixed case and control donor samples or studies with multiple timepoints per subject in each plate, which is a useful strategy to characterize intra-subject longitudinal progression but at the cost of increased interplate effects. In such scenarios, we find that skipping the median signal normalization step for all sample types except calibrators is an effective procedure to prevent plate bias issues. An alternative, valid strategy is to apply median signal normalization to the aggregate of all plates after Hyb.Cal normalization. However, it should be noticed that, as new datasets are added, data from previous runs would need to be renormalized retrospectively, which could pose difficulties e.g. in validating hypotheses derived from previously acquired datasets.

### Intraplate Variability using Calibrators

Typically, 5 to 7 replicates of calibrator samples, consisting of a common pooled sample of serum or plasma used across a large number of runs, are included in each plate. Per SomaLogic’s guidelines, these calibrators are utilized by design to normalize data across plates. In this Section, however, we will use them as probes to assess the intraplate variability of the SOMAscan Assay. For each plate and SOMAmer, we determined its CV over calibrator replicates as well as the ratio between the average calibrator RFU and the average buffer RFU; then, median values over all plates were computed.

**Figure 4:**
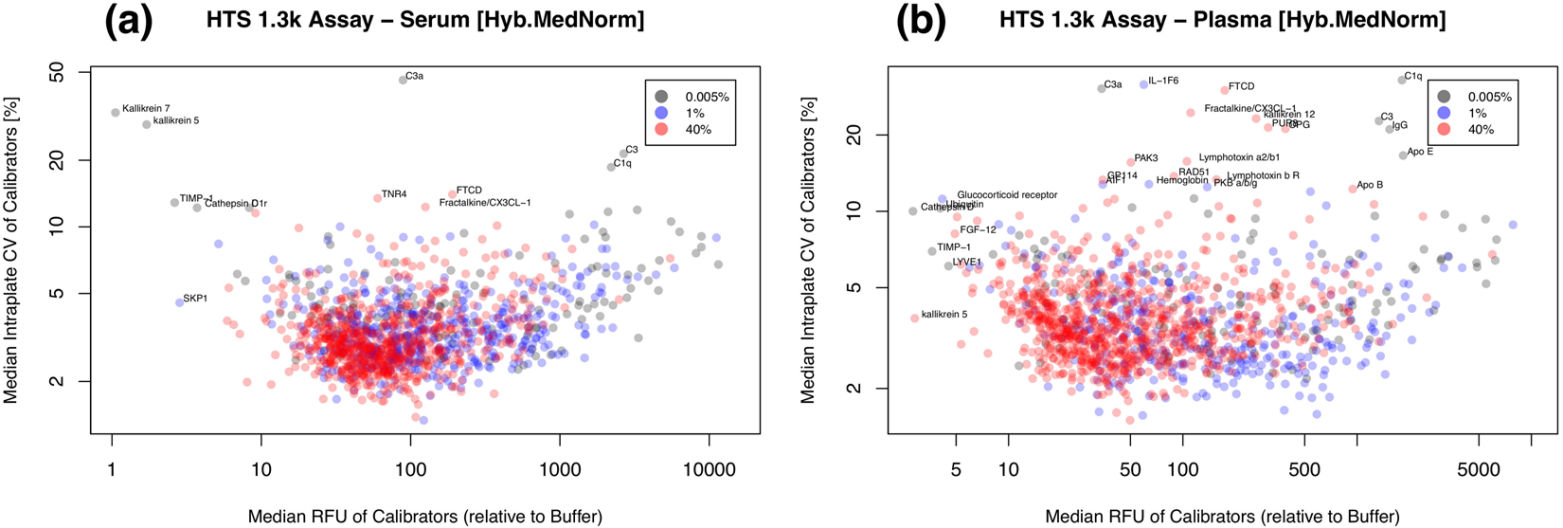
Median intraplate CV of calibrators as a function of the median intraplate RFU relative to buffer for (**a**) serum and (**b**) plasma. Data were generated with the HTS 1.3 k Assay and normalized by Hyb.MedNorm.

Figure 4(a,b) shows the median intraplate CV of calibrators as a function of the median intraplate RFU relative to buffer (i.e. the ratio *RFU* ^*Cal*^/*RFU* ^*Buffer*^) for serum and plasma, respectively, using data generated with the HTS 1.3 k Assay and normalized by Hyb.MedNorm. SOMAmer dilution groups are shown as well; notice that dilution group assignments depend on the biological matrix and may differ between serum and plasma. Labels indicate outlier SOMAmers. SOMAmer reagent characterization data has also been made available by SomaLogic^17^, where precision is reported from 3 QC samples run in triplicate over 3 assay runs (i.e. 9 total replicates for each of 3 QC samples) by averaging the precision across the QC samples. Supplementary Figure 4(a,b) shows the comparison of intraplate CVs in serum and plasma, respectively, in which a good agreement between our meta-analysis and SomaLogic’s reagent characterization is observed. Supplementary Figure 5 shows analogous results corresponding to the manual 1.1k Assay in serum. Summary statistics of these assays, including their breakdown by dilution group, are shown in Table 2. The overall technical variability of the assay is remarkably low, with a median intraplate CV in the ~3–4% range. As expected, the manual assay is more variable and this fact is more noticeable among SOMAmers in the 0.005% dilution group. Moreover, SOMAmer RFU expression is remarkably brighter than buffer for most SOMAmers. Among the SOMAmers with consistently poor performance, some are characterized, as expected, by low RFUs in the range of buffer (e.g. Kallikrein 5, Kallikrein 7, TIMP-1, SKP1, Cathepsin D) but it is also interesting to notice other, highly variable SOMAmers that are much brighter than buffer. Some of them are related to complement (e.g. C1q, C1r, C3, C3a) but we observe highly variable, highly expressed proteins with a variety of other functions as well (e.g. FTCD, Fractalkine/CX3CL-1, OPG, PAK3). From the above discussion on normalization procedures, it follows that Hyb.MedNorm is the most appropriate method to explore intraplate calibrator replicates. Supplementary Figures 6–7 present results obtained with Raw and Hyb data, respectively; despite larger CVs, as expected, the main qualitative features just described remain valid.

**Table 2:** Summary statistics: number of SOMAmers, median intraplate CV, and median RFU relative to buffer (based on calibrator data normalized by Hyb.MedNorm).

Dilution	Serum 1.3 k (HTS)			Plasma 1.3 k (HTS)			Serum 1.1 k (Manual)		
	*n*_SOMAmer_	CV[%]	RFU	*n*_SOMAmer_	CV [%]	RFU	*n*_SOMAmer_	CV [%]	RFU
All	1305	3.2	77	1305	3.6	53	1124	3.8	31
0.005%	130	4.5	163	124	4.7	170	120	6.9	76
1%	477	3.2	105	344	3.4	104	389	4.5	45
40%	698	2.9	61	837	3.5	40	615	3.0	24

As noted above, replicates of the same calibrator lot are included in a large number of assay runs; however, when that calibrator is depleted, SomaLogic must switch to a different calibrator lot. For the serum 1.3 k HTS Assay, two different calibrators were used (lot SL16689 in 11 plates and lot 160385 in 4 plates). Supplementary Figure 8 shows the correlation between calibrators for (a) the median intraplate CV and (b) the median intraplate RFU relative to buffer, which points out a high degree of consistency across calibrator lots. Similarly, for the plasma 1.3 k HTS Assay, three different calibrators were used (lot SL16485 in 4 plates, lot 160378 A in 3 plates, lot 160378 in 1 plate), whose pairwise correlations are shown in Supplementary Figure 9. All 11 plates for the serum 1.1k Manual Assay used the same calibrator, SL16689. Supplementary Figure 10(a,b) shows correlations of the median intraplate CV and RFU relative to buffer, respectively, of serum calibrator SL16689 between the 1.1k Manual Assay and the 1.3k HTS Assay over 1,061 shared SOMAmers. For 60 SOMAmer targets, the aptamer was replaced with another with better binding affinity and specificity; for 26 of them, the dilution group assignments were changed as well. Supplementary Figure 11(a,b) shows comparisons of these SOMAmer targets between the two assays; open symbols indicate SOMAmers that changed dilution group. This set of 60 SOMAmers does not correlate well, which is not surprising given the fact that their aptamer sequences are different; however, it is important to point this out when translating findings based on these 60 SOMAmers from 1.1k to the 1.3 k Assay.

Our web-based tool^15^ allows an interactive query of performance statistics (intraplate CV, total CV, and RFU relative to buffer) of individual SOMAmers by assay, which we expect to update regularly as our internal SOMAscan data repository grows. Moreover, as contributions from other SOMAscan deployment centers and individual users are received, SOMAmer performance measurements will become increasingly robust and accurate. Since this meta-analysis relies mostly on calibrator, QC and buffer samples, which are included in every plate and shared across a large customer base, we hope that data sharing will be encouraged for the benefit of the SOMAscan user community at large.

### Fold-Change Variability of Replicate Pairs

Thus far, we have characterized the SOMAscan Assay’s variability in terms of the coefficient of variation (CV). Qualitatively, it is obvious that a smaller CV reflects lower variability; in order to interpret CVs quantitatively, however, it is crucial to translate CVs into an operational index of variability on which we can perform rigorous statistical tests of significance. In serological assays, a two-fold disparity between repeated measurements has been widely regarded as the upper limit on an assay’s acceptable variability^18^. Wood^19^ showed the mathematical relationship between the frequency of fold-change ($FC$) disparities above the two-fold benchmark, *p*(*FC* ≥ 2), and the size of the standard deviation of repeated assay measurements, under the assumption that measurements are log-normally distributed. Building on Wood’s seminal work, Reed *et al*.^20^ derived the mathematical relationship between the measured CV and the probability to observe fold-changes above any arbitrary *FC* value (i.e. not just limited to two-fold differences) and for sets of replicates of size *n*_*rep*_ ≥ 2 (i.e. not just limited to duplicates). These results have two important applications: (i) In studies with paired samples (e.g. before and after a medical intervention, and/or involving disease progression), to quantitatively assess the statistical significance of the observed differences by comparing them to null model references; and (ii) in studies with technical and/or biological replicates, to assess whether the assay’s observed variability is within the expected variability established on the basis of larger data repositories within or across laboratories.

The probability that two replicate measurements differ by a fold-change equal or larger than *FC* is given by^20^

6 $$p(FC)=2{\rm{\Phi }}\{\frac{-{\mathrm{log}}_{e}(FC)}{\sqrt{2{\mathrm{log}}_{e}({(CV/100)}^{2}+\mathrm{1)}}}\},$$

where Φ is the cumulative standard normal distribution function and the fold-change is restricted to *FC* ≥ 1, without loss of generality.

**Figure 5:**
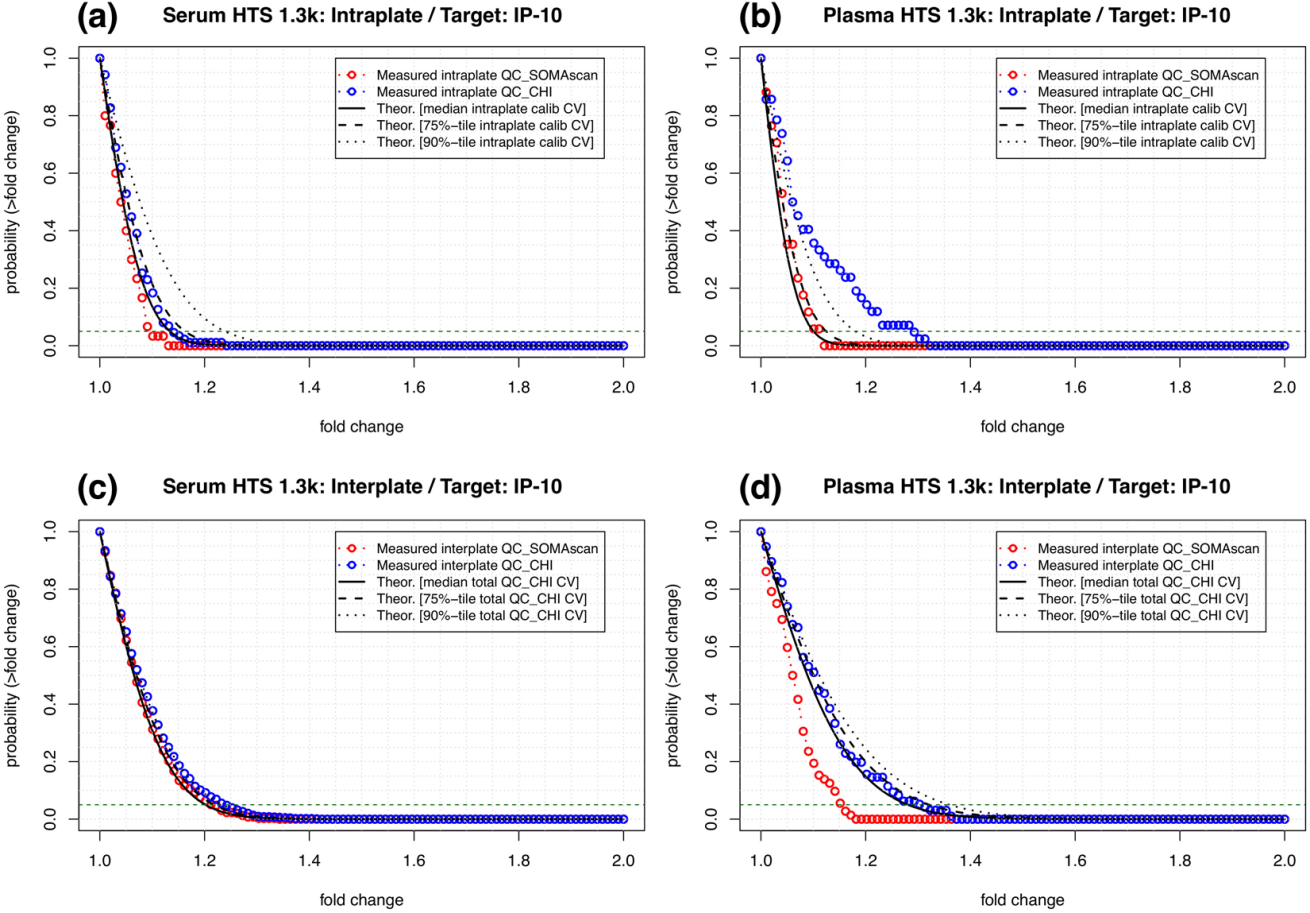
Probability that two replicate measurements of IP-10 will differ by a factor larger than a given fold change. Theoretical estimates (based on Eq. (6)) are compared to pairs of replicates among QC_SOMAscan and QC_CHI quality control samples. Top panels: Intraplate probability in (**a**) serum and (**b**) plasma. Bottom panels: Interplate probability in (**c**) serum and (**d**) plasma. Data were generated with the HTS 1.3k Assay and normalized by (**a,b**) Hyb.MedNorm and (**c**,**d**) Hyb.MedNorm.Cal, respectively.

For the sake of discussion, let us first focus on *Interferon gamma-induced protein 10* (IP-10), also known as *C-X-C motif chemokine 10* (CXCL10), which is a pro-inflammatory cytokine associated with multiple diseases. Figure 5(a,b) shows intraplate results for serum and plasma, respectively, using Hyb.MedNorm normalization. Theoretical curves obtained from Eq. (6) using the median, 75^th^ and 90^th^ percentiles of the intraplate CV of calibrators are compared to the measured fold-change frequency distributions of intraplate pairs of replicates among QC_SOMAscan and QC_CHI quality control samples. It is important to emphasize that the theoretical curves depend on just one parameter (i.e. the CV), which was determined from calibrators, i.e. independently from the quality control samples shown. Figure 5(c,d) shows interplate results for serum and plasma, respectively, using Hyb.MedNorm.Cal normalization. It should be noticed that, because calibrators are used to normalize the data across plates, we cannot use them to assess the total CV. Instead, we obtained the theoretical curves based on Eq. ((6)) by using the total CV of the QC_CHI bridge sample. In order to obtain a range of CV estimates, we generated *n*_*b*_ = 1000 bootstrapped sets of bridge samples and determined the CV within each set; then, we obtained the median, 75^th^ and 90^th^ percentiles over the distribution of *n*_*b*_ CV values. Similarly to the intraplate case above, these theoretical curves are compared to the measured fold-change frequency distributions of interplate pairs of replicates among QC_SOMAscan and QC_CHI quality control samples. In both serum and plasma, the observed variability is within the theoretical expectations. Supplementary Figure 12(a,b) shows analogous results for the Manual 1.1 k Assay in serum. Lacking an interplate bridge sample such as QC_CHI, we used 29 interplate duplicates that were measured from samples obtained from early-stage non-small-cell lung cancer patients and heavy smoker donor controls.

**Figure 6:**
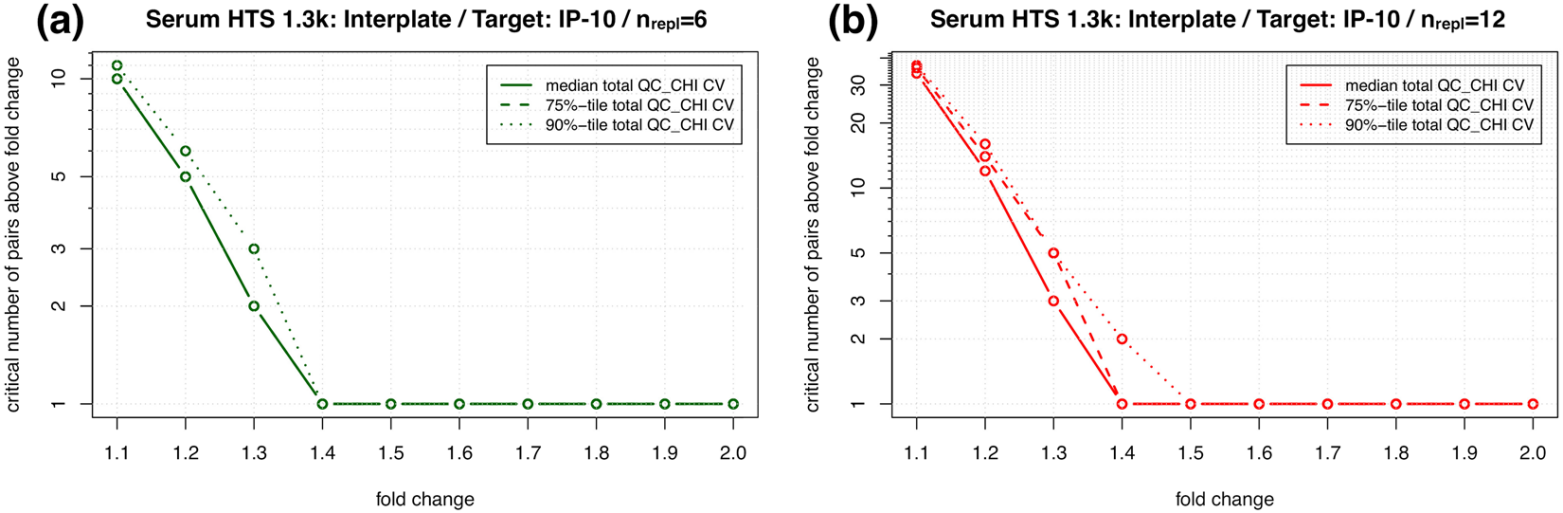
Critical number of IP-10 pairs above fold change in serum for different interplate replicate numbers: (**a**) *n*_*repl*_ = 6 and (**b**) *n*_*repl*_ = 12. If the number of pairwise fold change ratios from a study is equal or larger than these critical values, the assay’s technical variability is larger than expected based on our data repository.

Our web-based tool^15^ provides interactive access to intra- and interplate probability distributions of fold-change variability of individual SOMAmers by assay. As pointed out above, one application of these results is to lay out null model expectations of technical variability against which to assess the statistical significance of an observed fold-change. For instance, following the recent finding that plasma IP-10 may serve as an early biomarker for anti-TB chemotherapy response^21^, let us assume for the sake of argument that one patient experiences a decrease of 20% in IP-10 during treatment. Is this decrease statistically significant? In this case, we know that a 20% decrease corresponds to fold-change *FC* = 1.25. If the pre- and during-treatment plasma samples are measured in different plates, Fig. 5(d) shows that the two-tailed probability of an interplate fold-change equal or larger to the observed one is ${p}_{two-tailed}\simeq 0.15$ (using the theoretical prediction based on the 90^th^ percentile of the total QC_CHI, for a conservative approach). The probability of observing a decrease of 20% or larger by chance is thus ${p}_{one-tailed}\simeq 0.075$, which is too large to claim statistical significance under the usual *p* < 0.05 criterion. If, on the other hand, for another patient we measure a 40% decrease in IP-10, which corresponds to *FC* = 1.67, Fig. 5(d) tells us that such an observation is strongly significant, leading us thus to infer that this patient mounted an effective anti-TB response. Similar considerations can naturally also be applied to full cohorts, as for instance in the context of clinical trials. In a paired trial of size *n*_*trial*_, the two-tailed expected number of participants with effects larger than *FC*_*trial*_, a pre-established endpoint of the trial, is ${n}_{trial}\times p(F{C}_{trial})$. By observing an excess from this expectation, which is solely based on the assay’s technical variability, a measurable effect of the intervention would be confirmed. For paired study designs lacking a pre-established fold-change threshold, this value can be obtained from the condition *p*(*FC*_*thres*_) = 0.05. The number of observed fold-change pairs above *FC*_*thres*_ is then to be compared to the null model expectation of 5% of the cohort size. The case of unpaired study designs is discussed below. A second application of this framework is as a quality control tool to assess whether the assay variability measured in a laboratory setup is within the expected range. The reference variability is here provided by our meta-analysis, but other settings are possible (e.g. a laboratory testing the assay’s variability against its own internal reference, a multi-center study in which different laboratories must confirm that their SOMAscan setups are of comparable technical performance, or a SOMAmer-based test calibration in a clinical setting). Assuming that *n*_*repl*_ replicates are measured, the question to address is: How many replicate pairs may exhibit fold-change differences above a given fold-change threshold *FC*_*thres*_ solely based on the expected technical variability? We define *critical value* as the number of replicate pairs above a given fold-change threshold that would correspond to less than 5% chance for the assumed CV. Following the approach put forth by Reed *et al*.^20^, Monte Carlo simulations were used to obtain the critical value as a function of fold-change. Focusing again on IP-10 as our running example, Fig. 6 shows the critical number of pairs above fold change in serum for different interplate replicate numbers: (a) ${n}_{repl}=6$ and (b) *n*_*repl*_ = 12. For instance, let us assume that a laboratory performs quality control with *n*_*repl*_ = 6 and determines all 15 pairwise fold change ratios. If they observe 3 or more of their IP-10 ratios to lie above fold change 1.3, it is to be concluded that their technical variability is larger than the one expected based on our data repository, which might be due to the assay’s setup, the laboratory’s standard operating procedures, or due to other sources of variation unaccounted for. Our web-based tool^15^ provides interactive access to intra- and interplate critical value distributions for individual SOMAmers by assay, where a drop-down menu allows the user to select the number of replicates. For unpaired studies, we can state the null model hypothesis (*H*_0_: no difference between pre- and post-intervention) by considering all pre- and post-intervention samples as replicates. Assuming *n*_*repl*_ = *n*_*pre*_ + *n*_*post*_ and comparing all *n*_*pair*_ = *n*_*repl*_ × (*n*_*repl*_ − 1)/2 pairwise ratios against the fold-change threshold (which, as above, can be determined from the condition $p(F{C}_{thres})=0.05$), the observed number of pairs above *FC*_*thres*_ is to be compared to the null model expectation of 0.05 × *n*_*pair*_.

### Stability of Healthy Subject Baselines: A Case Study

In a typical study, multiple sources of variability are present; intraplate assay, plate-to-plate, and batch-to-batch effects, which represent different layers of technical variability, appear intertwined with intra- and inter-subject as well as intervention effects, which correspond to different sources of biological variability. In most applications, the former are nuisance factors and the latter represent effects of interest. In order to disentangle technical from biological effects, a study design should include control and replicate probes to address each layer of variability, which is a costly procedure that significantly constrains the room available to investigate new biology. In this Section, we show how our extensive technical characterization of the SOMAscan assay can be leveraged to assess relevant biological effects while minimizing the need for technical replicates.

In order to illustrate these ideas, let us consider a case study on the variability of healthy human subject sera at baseline. Details about the study design and samples can be found in Tsang *et al*.^22^. For this study, we used three timepoints per donor at days -7, 0, and 70 (after receiving an H1N1 influenza vaccine) in a cohort of size *n* = 14. This experiment was carried out via a single plate run in the HTS 1.1 k Assay. The comparisons of calibrator CV and RFU relative to buffer between this single-plate HTS run and the 11 runs with the Manual 1.1 k Assay are shown in Supplementary Figure 13. Based on gene expression microarrays and deep phenotyping 15-color flow cytometry, it has been previously shown that by day 70, measurable molecular and cellular markers were similar to pre-vaccine levels^22^, thus these could be considered three independent measures of healthy donor baseline timepoints. The question to address is the following: Which analytes and donors exhibit biologically stable baselines?

**Figure 7:**
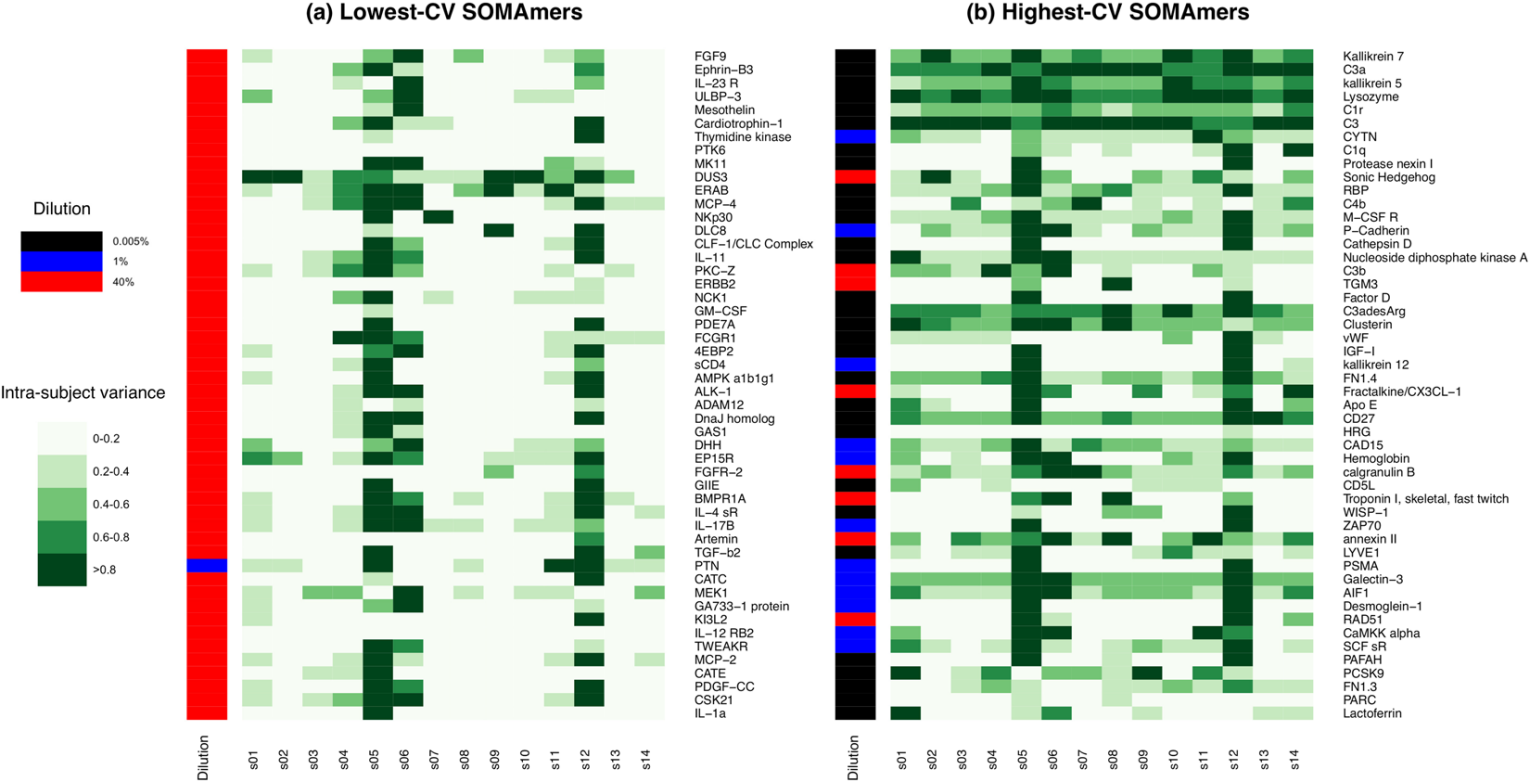
Top-50 SOMAmers of (**a**) lowest and (**b**) highest technical CV were individually assessed for biological intra-subject baseline variability across a cohort of 14 healthy subjects. In different shades of green, the heatmaps display, for each SOMAmer (row), the ratio between the variance of the expanded data associated to one subject and the variance of all subjects combined. Sidebars to the left of each panel display the SOMAmers’ dilution groups.

This scenario, which is typical of many studies, presents us with the challenge that the number of baseline samples per donor is too small to perform a reliable assessment of stability. However, we can leverage our knowledge of the assay performance to expand the dataset, as follows. For the *α*th SOMAmer, we have a technical CV value of reference, *CV*_*α*_, which was obtained from the median intraplate CV of calibrators in the Manual 1.1k Assay. Notice that, here and throughout, CV refers to the technical coefficient of variation, which has been established using calibrator samples; this is not to be confused with intra-subject (across timepoints) or inter-subject (across cohort) variability. For the *i*th subject/timepoint sample, this SOMAmer was measured as *RFU*_*iα*_. Following the usual assumption of log-normally distributed RFUs, we will assume that the distribution of *log*_*e*_(*RFU*) measurements representative of this SOMAmer/sample pair is given by a Gaussian distribution centered at *μ* = log_*e*_(*RFU*_*iα*_) and with standard deviation $\sigma =\sqrt{{\mathrm{log}}_{e}((C{V}_{\alpha }/{\mathrm{100)}}^{2}+\mathrm{1)}}$ ^20^. By randomly generating *n*_*rdm*_ = 1000 numbers from this distribution, we effectively expand the single subject/timepoint sample into a dataset amenable to further statistical analysis. In particular, we computed, for each SOMAmer, the ratio between the variance of the expanded data associated to one subject and the variance of all subjects combined, which is shown in Fig. 7(a,b) in two panels for the top-50 SOMAmers with lowest and highest technical CV, respectively.

**Figure 8:**
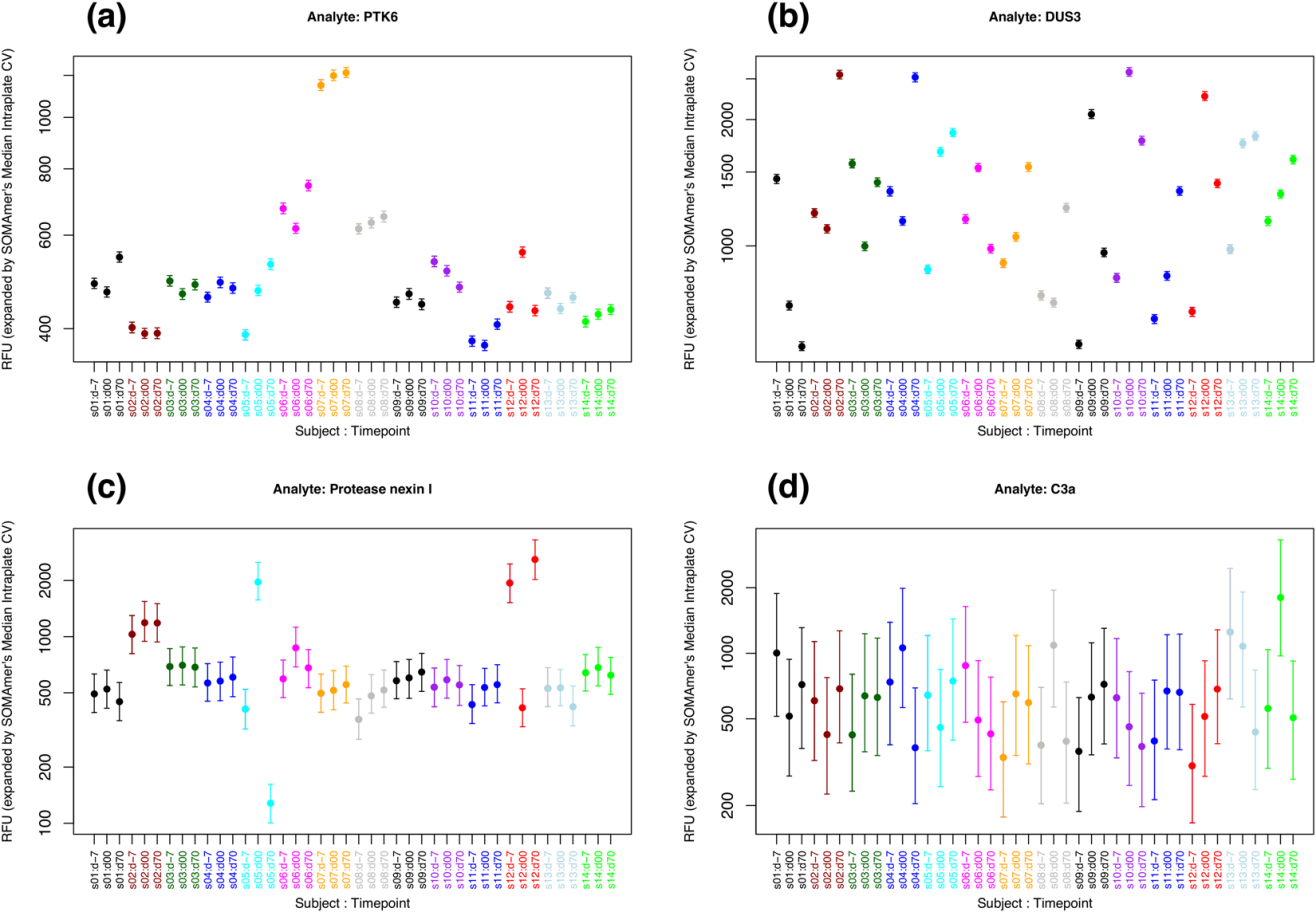
Intra- vs inter-subject variability of expanded subject/timepoint datasets for selected SOMAmers: (**a**) PTK6 (CV = 1.38%), (**b**) DUS3 (CV = 1.45%), (**c**) Protease Nexin I (CV = 14.6%), (**d**) C3a (CV = 39.2%). Vertical bars show the 5^th^ to 95^th^ percentile range of the expanded datasets.

We observe that subjects “s05”, “s12”, and, to a somewhat lesser extent, “s06”, appear highly variable for most SOMAmers across both panels. Moreover, SOMAmers in the high-CV group appear significantly more variable than SOMAmers in the low-CV group, as expected. In order to explore this further, Fig. 8 shows examples for individual SOMAmers; for each subject/timepoint sample, vertical bars show the 5^th^ to 95^th^ percentile range of the expanded dataset. Panel (a) shows one example of a SOMAmer with low technical CV (PTK6, CV = 1.38%) and stable intra-subject baseline. In contrast, Panel (b) shows a case of a SOMAmer with low technical CV (DUS3, CV = 1.45%) but unstable intra-subject baseline, which implies that intra-subject variability is comparable to subject-to-subject variability inherently due to the biology associated to this analyte in serum. Panel (c) displays the opposite case of a SOMAmer with high technical CV (Protease Nexin I, CV = 14.6%) but significantly stable intra-subject baselines for most donors; this is a case where the limiting factor for intra-subject stability appears to be technical rather than biological. Finally, Panel (d) shows a SOMAmer with high technical CV (C3a, CV = 39.2%) where no stability is apparent; the large technical variability, however, precludes any conclusions regarding the underlying biology.

In order to further characterize the stable analytes, we filtered the list by requiring that our stability measure (i.e. the intra-subject variance relative to the variance of all subjects combined) be ≥85% for 12 or more donors out of the 14 total (i.e. ≥85% of the cohort). The filtering procedure yielded 215 stable SOMAmers. Supplementary Table 1 provides a summary of our donor/analyte stability measurements for all 1124 SOMAmers in the 1.1 k Assay, where the stable SOMAmers are indicated. Subsequently, we performed a feature set enrichment analysis against the blood transcriptional modules (BTMs) from Li *et al*.^23^ and Chaussabel *et al*.^24^. After mapping SOMAmer IDs into Entrez gene symbols and removing those with no module assignments, we were left with 130 stable analytes out of 756. Table 3 shows the top hits from enrichment analysis via hypergeometric tests, which points to modules associated with signaling in T cells as well as modules associated with plasma cells, B cells, and immunoglobulins. Stable analytes for each module, as indicated by superscripts on the Table, are: (a) TNF, TNFRSF4, TNFRSF9, FASLG, IL2RA; (b) TNFRSF17, IGHG1, IGHD, IGJ; and (c) TNFRSF17, IGHG1, IGHD, IGHM. Similarly, unstable analytes for each module are: (d) GZMB, IFNG; (e) CD22; (f) CD27; and (g) GZMB, IFNG, TNFRSF18. Notice, however, that, due to the large number of modules tested, none of the hits remains statistically significant after false discovery rate (FDR) multiple testing adjustment of p-values.

**Table 3:** Blood transcriptional modules enriched for intra-subject stable analytes at baseline.

ID	annotation	stable	unstable	stable total	total	p-value	adj. p-value (FDR)
LI.M35.0	signaling in T cells (I)	5^a^	2^d^	130	756	0.0022	0.74
LI.M156.0	plasma & B cells, Ig’s	4^b^	1^e^	130	756	0.0036	0.74
LI.M156.1	plasma & B cells, Ig’s	4^c^	1^f^	130	756	0.0036	0.74
LI.M35.1	signaling in T cells (II)	5^a^	3^g^	130	756	0.0051	0.77

Several of the stable analytes involved in these modules belong to the tumor necrosis factor (TNF) superfamily, which regulates normal functions such as immune responses, haematopoiesis, and morphogenesis, but have also been implicated in tumorigenesis, transplant rejection, septic shock, viral replication, bone resorption, rheumatoid arthritis, and diabetes^25^. In the context of T cell signaling, the 19 ligands and 30 receptors of the TNF superfamily lead to a complex web of interactions, some of which are known to positively regulate T-cell responses and mediate crosstalk between T cells and other cell types^26^. The stability of these analytes in human serum supports theurapeutic strategies to block or promote TNF superfamily interactions to modulate the immune response. Furthermore, we also observe multiple hits involving immunoglobulin superfamily domains. Secreted immunoglobulins mediate the effector phase of humoral immunity, which results in the elimination of bound antigens. Here, we find stable intra-subject SOMAmer concentrations associated with immunoglobulins of different types (IgG, IgD, IGM) and the J chain (IGJ), which is a protein component of IgM and IgA that enables their secretion into mucosa. These observations indicate that the humoral immune response mediated by circulating immunoglobulin is tightly regulated.

## Discussion

In this paper, we presented an extensive meta-analysis of the current 1.3 k SOMAscan Assay in serum and plasma, as well as the former 1.1 k Assay in serum. We covered a variety of important topics that pertain to downstream data processing and interpretation.

Our analysis started by examining different normalization procedures. We found that the median normalization step, routinely performed per SomaLogic’s guidelines, is useful when applied to an homogeneous group of samples that is not expected to vary significantly from plate to plate (e.g. calibrators and QC samples) or in the context of single-plate runs. However, when used on heterogeneous sample groups (e.g. mixed case and control biological samples, especially in the case of plates with multiple timepoints per subject), the median normalization step may have the unwanted effect of reducing intraplate variance at the cost of increasing interplate effects. We illustrated this point with an analysis of samples from a multiplate longitudinal vaccination study based on an Adenovirus Type 4-Influenza H5 recombinant vaccine. As an alternative normalization procedure, we propose to skip the median normalization step except for calibrators. Batch effect removal methods, which have mostly been explored in the context of oligonucleotide microarrays^27–29^ and, to a lesser extent, in other proteomics assays^30,31^, are certainly an area that deserves further, in-depth investigation in the context of SOMAscan data.

By performing an intraplate meta-analysis of calibrator replicates, we obtained the CV and RFU relative to buffer for each SOMAmer in the assay. The overall technical variability of the assay was remarkably low, with a median intraplate CV in the ~3–4% range. The serum Manual assay showed an overall CV larger by ~20% relative to the serum HTS assay. As expected, dilution appeared to play a significant role in the variability of SOMAmers: The 0.005% dilution group had significantly larger CVs than the other groups. Roughly about one to two dozen SOMAmers showed unusually large CVs; some of them appeared to be related to complement, but others had a variety of other functions. This group of outliers was not associated with below-limit-of-detection issues; indeed, most of the SOMAmers, regardless of CV, were expressed at RFUs remarkably larger than buffer. The RFU relative to buffer appeared to be a very consistent measure across different calibrators and quality control samples. Similar qualitative features were observed for HTS and Manual assays, 1.3k and 1.1k, serum and plasma, and raw data as well as data normalized using different procedures. In this sense, the overall assay performance appeared to be quite robust.

In order to interpret CVs quantitatively, we implemented a theoretical model of statistical variability^20^ that provided probability distributions of technical replicate fold-changes. We discussed two applications of these results. On the one hand, fold-change probability distributions lay out null model references (based on the assay’s technical variability) to quantitatively assess the statistical significance of differences observed in studies with paired samples (e.g. in the context of medical interventions). On the other hand, as a quality control tool, critical value distributions determine whether the assay’s observed variability is within the expected variability established on the basis of larger data repositories within or across laboratories.

We developed an approach to assess the stability of healthy subject baselines and determined the set of analytes that exhibit biologically stable baselines after technical variability was factored in. This approach, based on a method of data expansion, may be used in other contexts in which the number of available replicate measurements is too small to perform a reliable de novo statistical analysis. Furthermore, we provided a list of stability measurements for all SOMAmers in the 1.1 k Assay, which represents a valuable reference for future studies.

Finally, we are making available an interactive web-based tool^15^ jointly released with this paper, which allows SOMAscan users to query our meta-analysis results on individual SOMAmers by choosing the appropriate assay and matrix. The tool provides performance statistics (intraplate CV, total CV, and RFU relative to buffer), fold-change probability distributions for intra- and interplate replicates, and critical value distributions for sets of intra- and interplate replicates. Moreover, an upload form is made available for users to share their raw data files for back-end processing. Since this meta-analysis relies mostly on calibrator, QC, and buffer samples, which are included in every plate and shared across a large customer base, it is our aim to encourage data sharing for the benefit of the SOMAscan user community at large. This resource, together with other open source web tools available for SOMAscan data QC and analysis^32,33^, enhances the ability for users to explore, analyze, and interpret their data. Hopefully, this initiative will stimulate further efforts towards regularly updated, high quality repositories with data sharing, reproducibility, and reusability as ultimate goals.

## Electronic supplementary material

Supporting Information to Assessment of Variability in the SOMAscan Assay

Supplementary Dataset 1
