# Supplementary material for: Assessment of Variability in the SOMAscan Assay
Source: Sci Rep. 2017 Oct 27;7:14248. doi: 10.1038/s41598-017-14755-5 (PMC5660188; doi:10.1038/s41598-017-14755-5)
Supplement: Supplementary file 1 — Supporting Information to Assessment of Variability in the SOMAscan Assay [file 41598_2017_14755_MOESM1_ESM.pdf]

Supporting Information to "Assessment of Variability in the SOMAscan Assay"

Julián Candia,[*,†] Foo Cheung,[†] Yuri Kotliarov,[†] Giovanna Fantoni,[†] Brian Sellers,[†] Trevor Griesman,[‡] Jinghe Huang,[‡] Sarah Stuccio,[‡] Adriana Zingone,[¶] Bríd M. Ryan,[¶] John S. Tsang,[†,§] and Angélique Biancotto[†]

†*Trans-NIH Center for Human Immunology, Autoimmunity, and Inflammation, National Institutes of Health, Bethesda, MD 20892, USA*

‡*Laboratory of Immunoregulation, National Institute of Allergy and Infectious Diseases, National Institutes of Health, Bethesda, MD 20892, USA*

¶*Laboratory of Human Carcinogenesis, Center for Cancer Research, National Cancer Institute, National Institutes of Health, Bethesda, MD 20892, USA*

§*Systems Genomics and Bioinformatics Unit, Laboratory of Systems Biology, National Institute of Allergy and Infectious Diseases, National Institutes of Health, Bethesda, MD 20892, USA*

E-mail: julian.candia@nih.gov

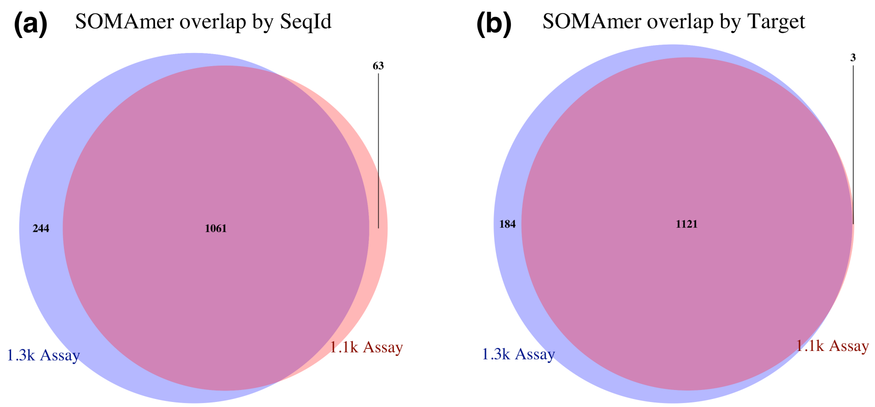

Supplementary Figure 1: Venn diagram comparisons between the 1.3k and 1.1k Assays based on (a) aptamer sequence ("SeqId") and (b) target analyte, respectively. For a total of 1061 SOMAmers, the aptamer sequence remained unchanged. However, for 60 SOMAmer targets, the aptamer sequence was modified to improve performance.

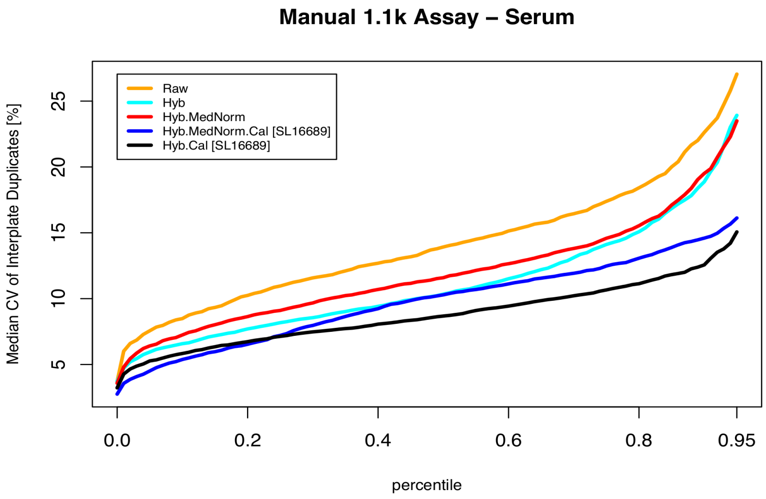

Supplementary Figure 2: Percentile distributions (across all SOMAmers) of the interplate CV obtained from 29 duplicates of serum samples from early-stage non-small-cell lung cancer patients and heavy smoker donor controls run manually in the 1.1k Assay.

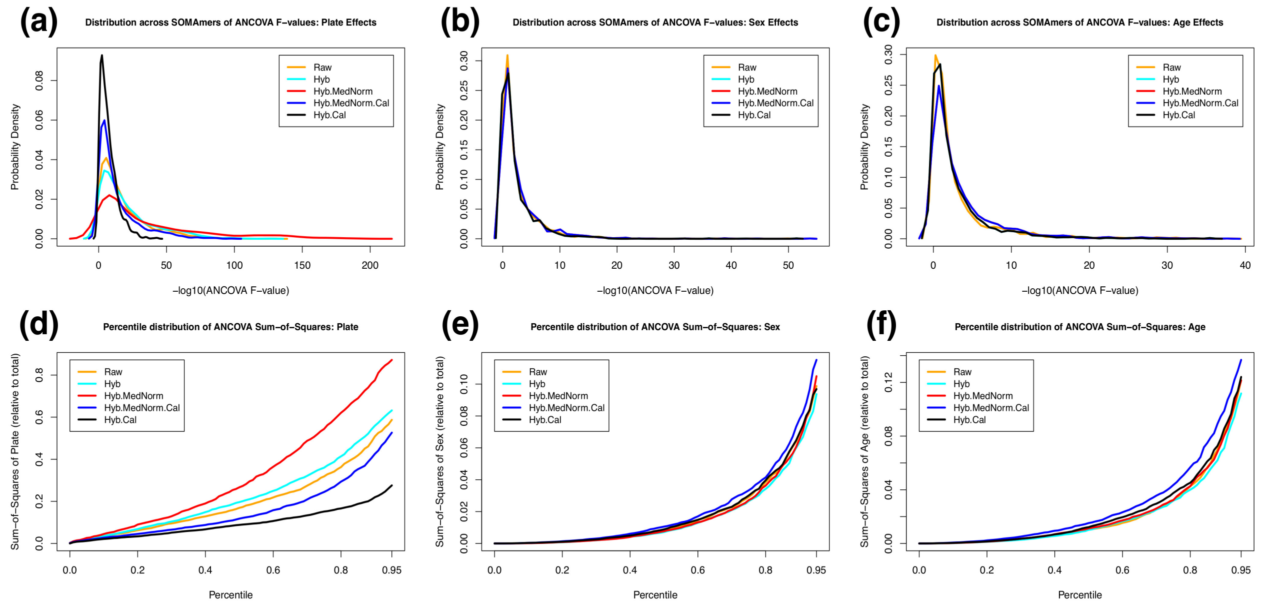

Supplementary Figure 3: Top panels: Distribution of ANCOVA F-values across all SOMAmers for (a) plate, (b) sex, and (c) age effects. Bottom panels: Percentile sum-of-squares distributions (across all SOMAmers) of (d) plate, (e) sex, and (f) age relative to the total SoS. Data were generated with the HTS 1.3k Assay.

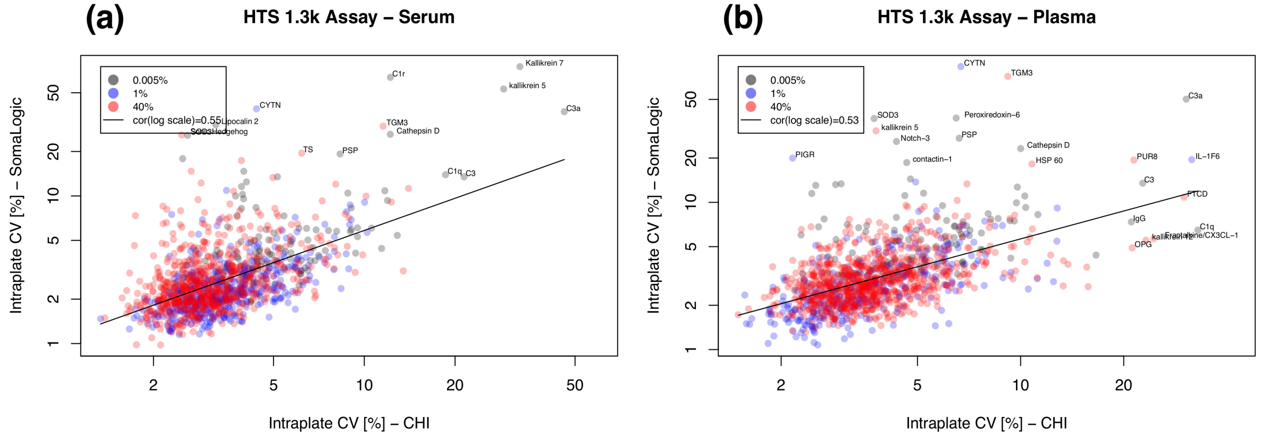

Supplementary Figure 4: Correlation of intraplate CV between our meta-analysis (CHI) and SomaLogic's reagent characterization for (a) serum and (b) plasma with the HTS 1.3k Assay. Data were normalized by Hyb.MedNorm.

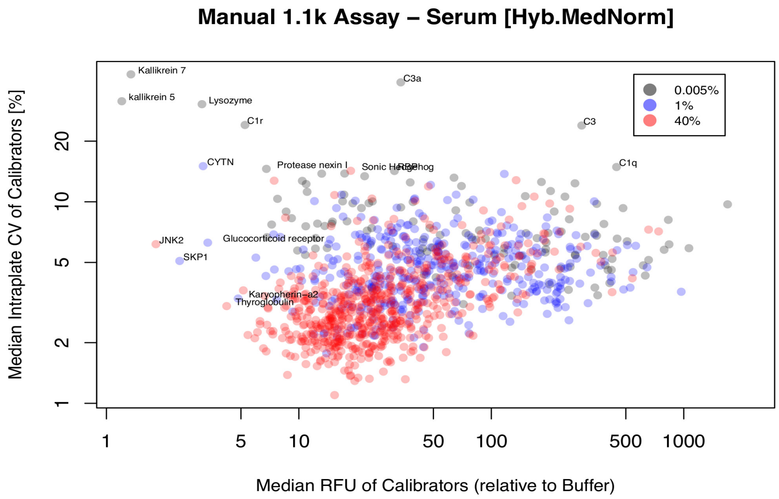

Supplementary Figure 5: Median intraplate CV of calibrators as a function of the median intraplate RFU relative to buffer. Data were generated from serum samples with the manual 1.1k Assay and normalized by Hyb.MedNorm.

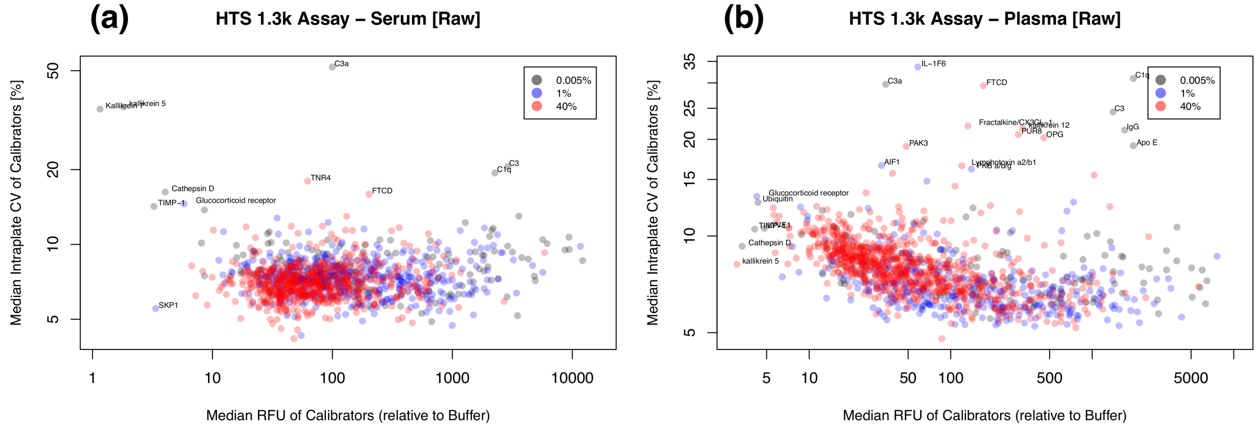

Supplementary Figure 6: Median intraplate CV of calibrators as a function of the median intraplate RFU relative to Buffer for (a) serum and (b) plasma. Data were generated with the HTS 1.3k Assay and were not normalized.

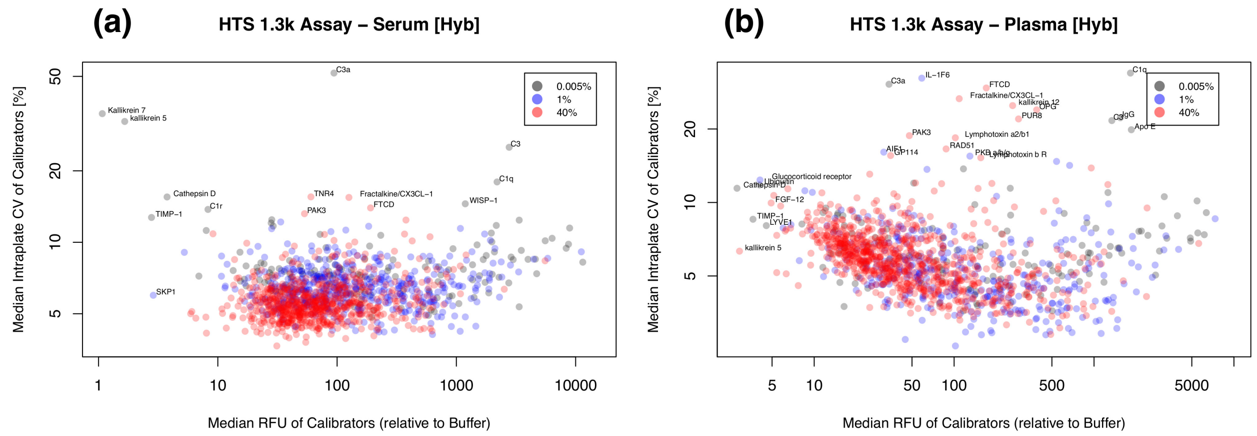

Supplementary Figure 7: Median intraplate CV of calibrators as a function of the median intraplate RFU relative to Buffer for (a) serum and (b) plasma. Data were generated with the HTS 1.3k Assay and normalized by Hyb.

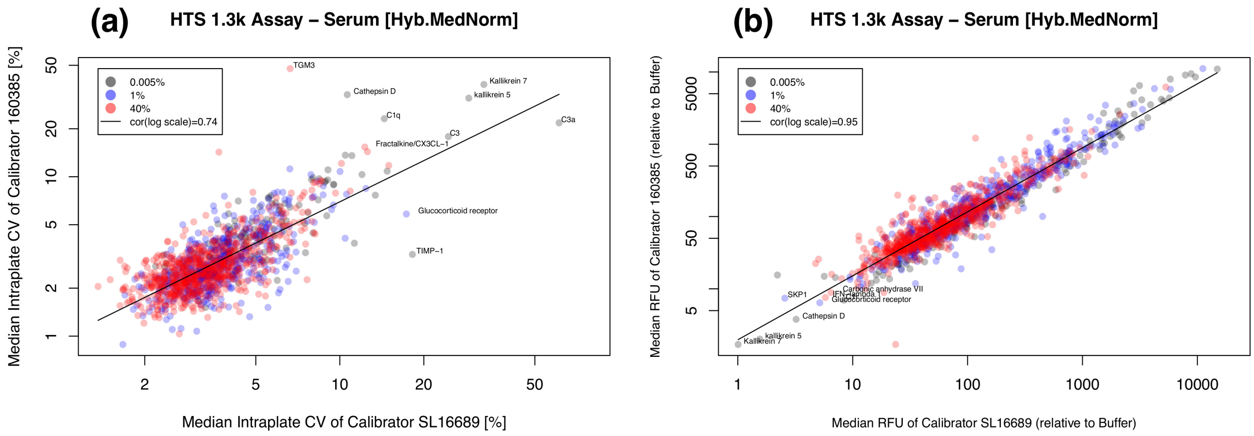

Supplementary Figure 8: Correlation between calibrators for (a) the median intraplate CV and (b) the median intraplate RFU relative to Buffer for runs generated with the serum 1.3k HTS Assay. Calibrator SL16689 was used in 11 plates and Calibrator 160385 in 4 plates. Data were normalized by Hyb.MedNorm.

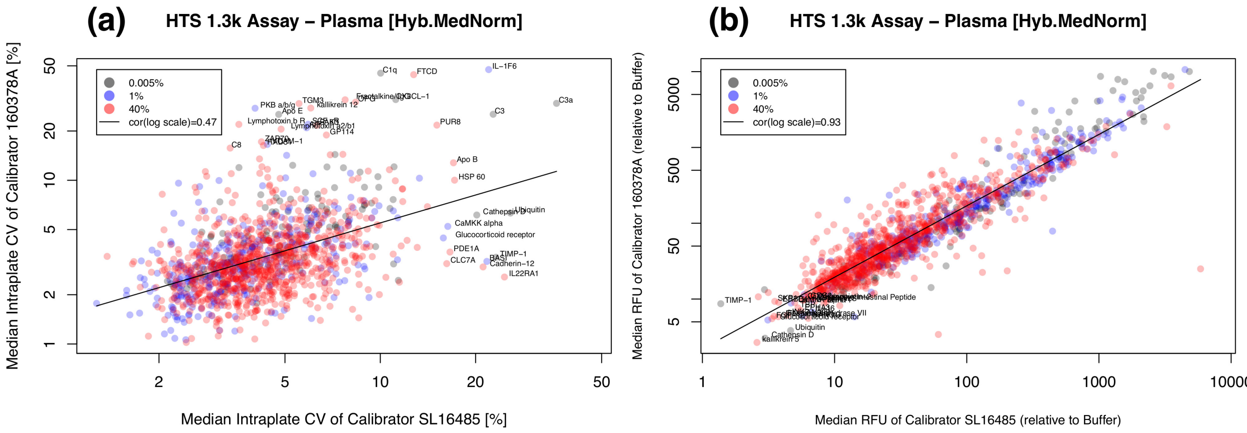

Supplementary Figure 9: Correlation between calibrators for (a) the median intraplate CV and (b) the median intraplate RFU relative to Buffer for runs generated with the plasma 1.3k HTS Assay. Calibrator SL16485 was used in 4 plates, Calibrator 160378A in 3 plates, and Calibrator 160378 (not shown) in 1 plate. Data were normalized by Hyb.MedNorm.

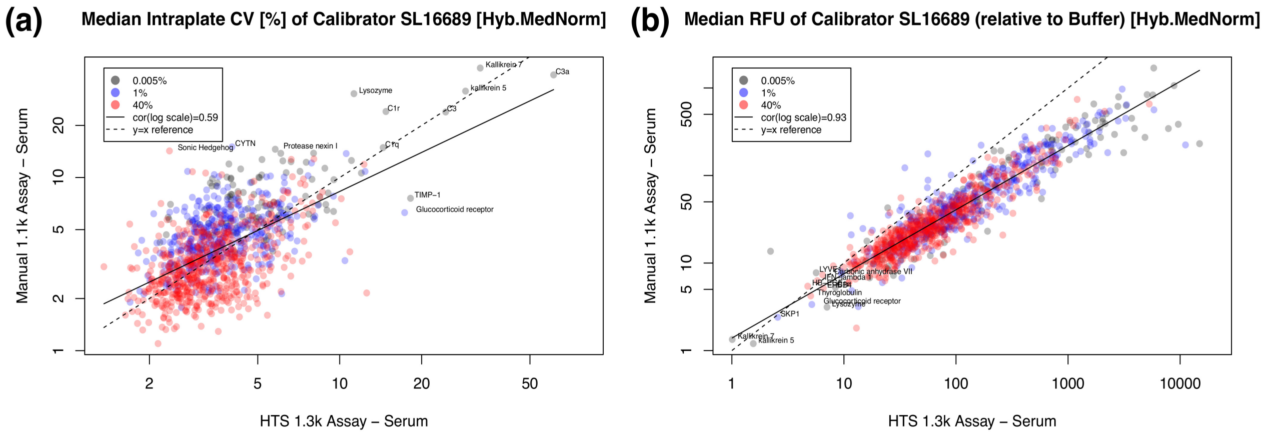

Supplementary Figure 10: Correlation between the 1.3k HTS Assay and the 1.1k Manual Assay over 1061 shared SOMAmers for serum calibrator SL16689: (a) median intraplate CV; (b) median intraplate RFU relative to Buffer. Data were normalized by Hyb.MedNorm.

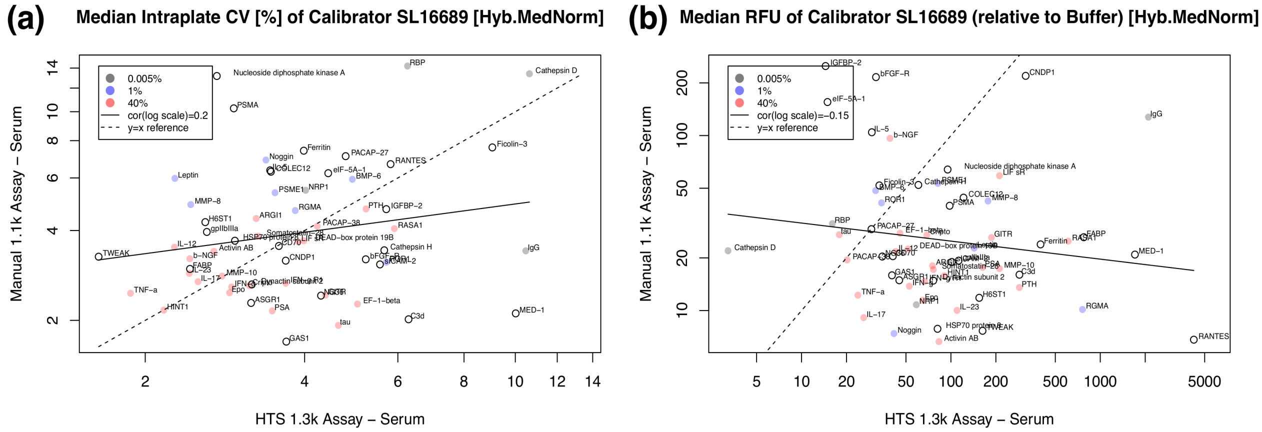

Supplementary Figure 11: Correlation between the 1.3k HTS Assay and the 1.1k Manual Assay over 60 SOMAmers with same target but different aptamer sequence for serum calibrator SL16689: (a) median intraplate CV; (b) median intraplate RFU relative to Buffer. Data were normalized by Hyb.MedNorm.

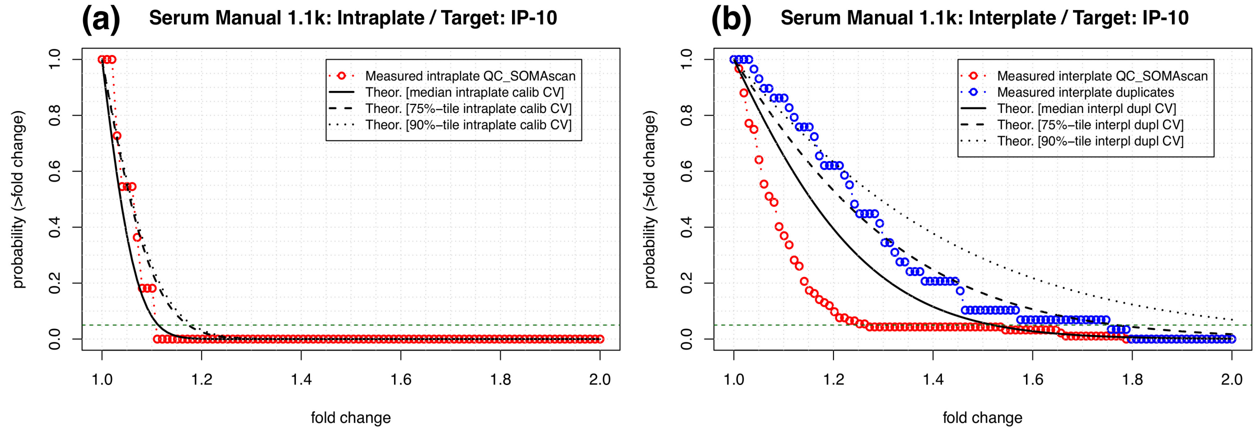

Supplementary Figure 12: Probability that two replicate measurements of IP-10 will differ by a factor larger than a given fold change. Theoretical estimates are compared to pairs of replicates among QC_SOMAscan quality control samples, as well as among 29 interplate duplicates from samples obtained from early-stage non-small-cell lung cancer patients and heavy smoker donor controls. Data were generated with the Manual 1.1k Assay and normalized by (a) Hyb.MedNorm and (b) Hyb.MedNorm.Cal, respectively.

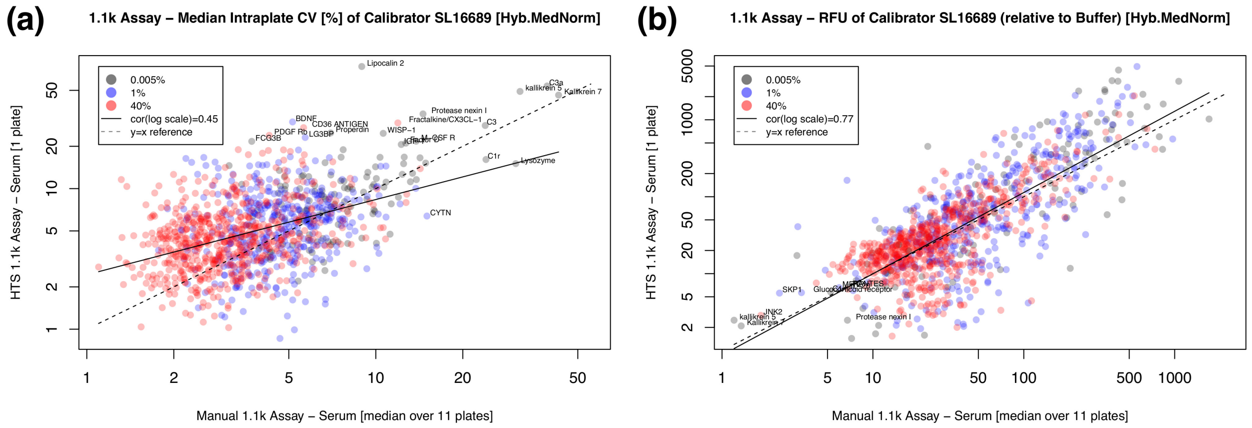

Supplementary Figure 13: Correlation between Manual and HTS runs for serum calibrator SL16689 in the 1.1k Assay: (a) median intraplate CV; (b) median intraplate RFU relative to Buffer. Data were normalized by Hyb.MedNorm.
